# What Is New in the Management of High-Risk Localized Prostate Cancer?

**DOI:** 10.3390/jcm12020455

**Published:** 2023-01-06

**Authors:** Mudassir Wani, Sanjeev Madaan

**Affiliations:** 1 Department of Urology, Swansea Bay University Health Board, Swansea SA6 6NL, UK; 2 Department of Urology, Darent Valley Hospital, Dartford DA2 8DA, UK; 3 Institute of Medical Sciences, Canterbury Christchurch University, Canterbury ME4 4UF, UK

The current Special Issue, in the Journal of Clinical Medicine, is dedicated to collecting high-quality research that mainly focuses on “Clinical advances in Prostate Cancer Treatments”.

Prostate cancer (PCa) was reported as being the third most commonly diagnosed malignancy by the World Health Organization (WHO) in 2020. Lung (2,206,771 cases; 11.4%) and colorectal cancer (1,148,515 cases; 10.0%) were the other two malignancies ahead of prostate cancer (1,414,259 cases; 7.3% of the total) [1]. However, in over 50% of the countries in the world (112 out of 185), it is the most diagnosed cancer. Prostate cancer incidence has been found to be associated with the Human Development Index (HDI), being more common in countries with high HDI as compared to countries with low HDI (37.5 vs. 11.3 per 100,000 people) [2]. PCa mortality is significantly different from its incidence rate. The highest mortality rates across the globe reveal 75.8 per 100,000 people in the Caribbean, 22.0 per 100,000 people in Sub-Saharan Africa, and 18.8 per 100,000 people in Micronesia/Polynesia [1].

More than 80% of prostate cancer patients in the developed world usually present with localized disease and, of these, 15% are recognized as being at high risk for cancer recurrence [3]. The definition of high-risk disease most accepted globally is one put forward by D’Amico et al. consisting of a PSA greater than 20 ng/mL, a clinical T stage of at least cT2c, or a Gleason score of at least 8 [3]. This group of patients has a high risk of biochemical recurrence (BCR) estimated to be 70% in 5 years. Additionally, this group also has a significantly high risk of metastatic disease as well as mortality related to prostate cancer with approximately 85% dying within 10 years post-diagnosis [4]. The treatment of these patients with single modality regimens such as radical prostatectomy or radiation therapy has proven to have a poor treatment response with high failure rates as well as more than 50% chances of both clinical and biochemical progressions at 5 years [5]. Most of these patients are offered a combination of radiotherapy with hormonal therapy, which is often considered an inadequate therapeutic approach and is associated with significant adverse effects.

Recent advances in prostate surgery in the form of robot-assisted radical prostatectomy (RARP) have provided a safe, standard, as well as oncologically effective surgical option in the management of patients diagnosed with high-risk localized and/or locally advanced prostate cancer. A recent retrospective study, evaluating the role of RARP in these patients, found that 188 patients (100%) remained biochemical recurrence-free (BCR) as well as clinical recurrence-free (CR) during the 1- and 3-year follow-up post-RARP [6]. More refinements in RARP, such as super-extended RARP, have been found to be useful in patients with either high-risk localized and/or locally advanced prostate cancer [7]. New extended risk-stratification of radical prostatectomy for non-metastatic high-risk prostate cancer, based on the European Multicenter Prostate Cancer Clinical and Translational (EMPaCT) research database, has been found to be helpful in determining cancer-specific survival (CSS) post-radical prostatectomy in these patients [8].

The optimal timing of post-radical prostatectomy radiotherapy (adjuvant radiation therapy versus early salvage radiation therapy) in high-risk PCa patients is still debatable. A recently published systematic review and meta-analysis have suggested that in men with localized or locally advanced prostate cancer, there is no improvement in event-free survival after adjuvant radiotherapy [9]. Further, the study recommends that early salvage treatment should be the preferred treatment option to spare many patients from radiotherapy and its associated adverse effects [9].

Systemic therapy in combination with radical prostatectomy is also being extensively studied. There has been a renewed interest in neoadjuvant hormonal treatment in high-risk prostate cancer, particularly with the introduction of androgen receptor signalling inhibitors (ARSIs) [10]. The commonly used agents include—Abiraterone, Apalutamide, Enzalutamide, and Darolutamide. These agents have been found to be better in comparison to conventional neo-adjuvant Androgen Deprivation Therapy (ADT) in terms of long-term oncological outcomes. Presently, post-radical prostatectomy’s use of adjuvant hormonal (ADT), as well as adjuvant chemo-hormonal therapy, remains controversial given the outcomes from several studies and trials [11]. On the other hand, in patients with high-risk localized prostate cancer, the combination of hormonal therapy with radiation therapy is often considered a standard of care [11]. Studies and trials have proven cancer-specific as well as overall survival benefits with the combination [11]. The STAMPEDE trial, a multiarm, multistage, randomized controlled trial investigated the effect of the Abiraterone addition to ADT for patients with newly diagnosed hormone-sensitive prostate cancer [12]. The two groups received ADT alone or ADT plus Abiraterone for a period of 2 years. The patients were mandated to receive definitive radiation therapy. In subgroup analyses, the overall survival was in favour of abiraterone plus ADT as compared with ADT alone, as determined by the nodal status, metastasis status, and planned radiation therapy [12]. In the case of localized prostate cancer, the addition of chemotherapy to radiation therapy has not been able to achieve outcomes to be considered a standard management strategy [12].

Novel modalities are being investigated in the management of high-risk localized PCa. One of the approaches is combining chemotherapy with photodynamic therapy (PDT). Recently, a new multifunctional theranostic molecule has been developed, it involves a combination of chemotherapy, imaging, and PDT for therapy against prostate-specific membrane antigen (PSMA) [5]. It involves the use of a PSMA ligand to deliver a potent microtubule inhibiting agent selectively and simultaneously, Monomethyl Auristatin E (MMAE), and a PDT agent, IR700, to prostate cancers [5]. In comparison with individual treatment with PDT or chemotherapy alone, this multifunctional molecule has shown selective uptake in PSMA-positive tumours with significant antitumor activity [5]. This study suggests that it can be used during surgery to help in identifying tumours using real-time fluorescence image-guided surgery (FIGS) and provide PDT as well as chemotherapy to kill any unresected cancer cells [5].

Immunotherapy is also being investigated in the management of high-risk localized and locally advanced PCa. Immunotherapy options include anti-PCa vaccines, e.g., Sipuleucel-T, and the use of immune checkpoint inhibitors (anti-CTLA-4 and anti-PD-1/PD-L1 monoclonal antibodies or antagonists) [13]. Immunologically, PCa is referred to as a “cold” tumour as it is not able to trigger a strong immune response, primarily due to reduced or a complete lack of T-cell infiltration [14]. Studies have revealed that myeloid-derived suppressor cells (MDSCs) play a vital role in PCa development as well as progression. This is encouraging and suggests their potential as a therapeutic target. Hence, the scope of immunotherapy in PCa may be widened by combination therapy such as immunotherapy and radiotherapy, targeting both the MDSCs and the tumour [14].

In conclusion, the continuous search for and development of novel approaches are essential for managing high-risk prostate cancer cases.
